# Thromboelastography combined with conventional coagulation parameters for developing a nomogram model to predict postpartum hemorrhage risk: A retrospective analysis

**DOI:** 10.12669/pjms.42.7.14405

**Published:** 2026-07

**Authors:** Yufang Zhang, Yanju Zhu, Haiyan Wang, Mengyuan Wang, Yanwei Guo

**Affiliations:** 1 Yufang Zhang, Department of Obstetrics and Gynecology, Affiliated Hospital of Chengde Medical College, Chengde 067000, Hebei, China; 2 Yanju Zhu, Department of Obstetrics and Gynecology, Affiliated Hospital of Chengde Medical College, Chengde 067000, Hebei, China; 3 Haiyan Wang, Department of Obstetrics and Gynecology, Affiliated Hospital of Chengde Medical College, Chengde 067000, Hebei, China; 4 Mengyuan Wang, Department of Obstetrics and Gynecology, Affiliated Hospital of Chengde Medical College, Chengde 067000, Hebei, China; 5 Yanwei Guo, Department of Obstetrics and Gynecology, Affiliated Hospital of Chengde Medical College, Chengde 067000, Hebei, China

**Keywords:** Coagulation parameter, Nomogram model, Postpartum hemorrhage, Thromboelastography

## Abstract

**Objectives:**

To construct a quantitative nomogram for predicting the risk of PPH in parturients by integrating thromboelastography(TEG) with conventional coagulation parameters [prothrombin time (PT), activated partial thromboplastin time(APTT), and fibrinogen(FIB)] to provide a clinical tool for early intervention.

**Methodology:**

A retrospective analysis was conducted on the clinical data of 500 parturients who delivered at Affiliated Hospital of Chengde Medical College between January 2021 and September 2025. Participants were divided into a PPH group (*n =* 85) and a non-PPH group (*n =* 415) based on whether PPH occurred. Collected the clinical characteristics, TEG parameters, and conventional coagulation parameters [thrombin time (TT), APTT, PT, FIB, D-dimer (D-D), and antithrombin-III (AT-III]) of the enrolled participants.

**Results:**

There were statistically significant differences in the comparison of the number of previous abortions, history of preterm birth, previous cesarean section,prior PPH, anemia, prolonged labor, placenta previa, placental abruption, placenta accreta, coagulation index (CI), FIB, D-D, and AT-III between the non-PPH group and the PPH group (all *P <* 0.05). Logistic regression identified previous cesarean section,previous PPH, anemia,coagulation index (CI), D-D and AT-III as independent risk factors for PPH (all *P <* 0.05), whereas FIB were protective factors (all *P <* 0.05). The bootstrap-based internal validation (1,000 resamplings) with an optimism-corrected bias of 0.007.

**Conclusion:**

The nomogram integrating TEG parameters with conventional coagulation parameters demonstrates excellent discrimination, calibration, and predictive accuracy for assessing PPH risk in parturients.

## INTRODUCTION

Postpartum hemorrhage (PPH) remains the leading cause of maternal mortality worldwide, particularly in resource-limited settings where early recognition and timely intervention are critical for improving outcomes.^1^ Despite continuous advancements in obstetric care, the incidence of PPH has shown an upward trend in recent years. In some cases, disease progression can be rapid, and conventional early-warning indicators often lack sufficient sensitivity.^2^ Conventional coagulation parameter testing is a common choice clinically, which generally involves prothrombin time (PT), activated partial thromboplastin time (APTT), fibrinogen (FIB), and D-dimer (D-D). However, these tests, focusing primarily on static, *in vitro* coagulation processes, cannot fully capture the dynamic *in vivo* interplay between coagulation and fibrinolysis.

This limitation is particularly evident in the hypercoagulable state of pregnancy, where their predictive performance is often suboptimal.^3^,^4^ Thromboelastography (TEG), a whole-blood assay for global coagulation monitoring, offers a dynamic assessment of the entire hemostatic process, including coagulation initiation, clot formation, fibrin cross-linking, and fibrinolysis. Key parameters, such as reaction time (R), maximum amplitude (MA), coagulation time (K), coagulation index (CI), and α-angle, provide comprehensive information that unveil the physiological coagulation status more accurately.^5^ As evidenced by existing research, TEG parameters possess superior predictive value for obstetric hemorrhage compared with traditional coagulation tests, particularly in detecting hypofibrinogenemia, platelet dysfunction, and hyperfibrinolysis.^6^

However, few studies have integrated TEG parameters with conventional coagulation parameters to establish a comprehensive predictive model for quantifying maternal PPH risk. Noticeably, nomogram is a visual and individualized prediction tool, enabling the generation of personalized risk scores by integrating multidimensional clinical variables. It has been widely applied in oncology and cardiovascular research.^7^,^8^ In this study, a PPH risk prediction nomogram was developed and validated based on the integration of prepartum maternal TEG parameters and conventional coagulation parameters. It is expected to construct a clinically practical early warning model to facilitate accurate identification and stratified management of high-risk parturients, thereby reducing the incidence of adverse outcomes associated with PPH.

## METHODOLOGY

This retrospective study was conducted by including 500 parturients who delivered at Affiliated Hospital of Chengde Medical College between January 2021 to September 2025. All data of the enrolled participants were collected from the Electronic Medical Record System of this hospital independently by two obstetric researchers who had received standardized training. The collected information included maternal age, body mass index (BMI) at delivery, gravidity, parity, number of abortions, history of preterm birth, gestational age at delivery, mode of delivery, previous cesarean section, prior PPH, anemia, prolonged labor, placenta previa, macrosomia, placental abruption, placenta accreta, and pregnancy-related comorbidities (gestational hypertension, gestational diabetes, and thyroid dysfunction).

### Ethical approval:

The study was approved by the Institutional Ethics Committee of Affiliated Hospital of Chengde Medical College (No.: CYFYLL2023527; Date: November 26, 2023), and written informed consent was obtained from all participants.

### Inclusion criteria:

- Women (20~45 years old) who received regular antenatal care and subsequently delivered at Affiliated Hospital of Chengde Medical College.
- Obtained the informed consent of participants’ families.
- Singleton pregnancy.
- Undergoing TEG and conventional coagulation testing before delivery.
- Gestational age ≥ 28 weeks (with the exclusion of preterm birth-related special bleeding risk factors and focusing on term and late-pregnancy deliveries).
- Delivery modes involving spontaneous vaginal delivery, assisted vaginal delivery (*e.g*., forceps or vacuum extraction), or cesarean section, with complete clinical documentation of intrapartum and 24-hour postpartum blood loss, allowing definitive determination of PPH occurrence.

### Exclusion criteria:

- Multiple gestations.
- Presence of congenital coagulation disorders, chronic hepatic or renal disease, hematologic disorders, or long-term use of anticoagulant therapy.
- Incomplete or missing clinical data.
- History of surgery, acute infection, major trauma, or medication that affected the coagulation significantly within one month before delivery.
- Tumors and other diseases

### Clinical grouping:

Participants were divided into a PPH group (*n =* 85) and a non-PPH group (*n =* 415). According to the diagnostic criteria outlined in the *Guidelines for Prevention and Treatment of Postpartum Hemorrhage (2023)*^9^, PPH was defined as blood loss ≥500 mL within 24 hours after vaginal delivery or ≥1,000 mL after cesarean section. The non-PPH group included women with blood loss <500 mL (vaginal delivery) or <1,000 mL (cesarean section) during the same period.

### TEG and Conventional Coagulation Parameter Testing:

Before delivery, blood samples were collected in all cases under fasting condition in the morning, each participant was subjected to the collection of 6 mL of peripheral venous blood into two tubes. One tube of the blood sample was analyzed using a TEG analyzer (Medcaptain, model: HaemaTx) to determine TEG parameters, including R, MA, K, CI, and α-angle. The other tube was used for conventional coagulation parameter (*e.g*., TT, APTT, PT, FIB, D-D, and AT-III) testing on an automated coagulation analyzer (Sysmex Corporation, models CS-5100 and CN-6000).

### Statistical analysis:

All statistical analyses were performed using SPSS 26.0. Continuous variables were expressed as mean ± standard deviation (). The Kolmogorov-Smirnov test was used to assess the normality of data distribution. Variables conforming to a normal distribution were compared using the independent-sample t-test, whereas non-normally distributed data were expressed as median (M [P25, P75]) and analyzed using nonparametric tests. Categorical variables were presented as counts and percentages (*n*[%]) and compared using the chi-square (χ²) test. Logistic regression analysis was employed to identify independent risk factors associated with PPH. Significant predictors were subsequently entered into the R software to construct a risk prediction nomogram. Internal validation of the model was performed using the bootstrap resampling method (1,000 iterations), and the bias-corrected was calculated to assess model discrimination. A calibration curve was generated to evaluate the agreement between predicted and observed probabilities, and the *Hosmer-Lemeshow* goodness-of-fit test was used to assess model calibration. The area under the receiver operating characteristic (ROC) curve (AUC) was calculated to evaluate the predictive performance of the model within the study sample. A *P*-value <0.05 was considered statistically significant.

## RESULTS

There were significantly statistical differences in the number of previous abortions, history of preterm birth, previous cesarean section, prior PPH, anemia, prolonged labor, placenta previa, placental abruption, placenta accreta, as well as in TEG parameters (i.e., CI) and coagulation parameters (*i.e*., FIB, D-D, AT-III) between the PPH and non-PPH groups (all *P <* 0.05). However, no statistically significant differences were found in the remaining data (all *P >* 0.05). Table-I.

**Table-I:** Comparison of clinical characteristics, TEG parameters, and conventional coagulation parameters between the PPH and non-PPH groups.

Parameters	PPH (n = 85)	Non-PPH (n = 415)	χ²/t/Z value	P-value
Age (years)			1.219	0.270
20-34	68(80.00)	352(84.82)		
>35	17(20.00)	63(15.18)		
BMI at delivery (kg/m²)	27.59±3.45	27.38±3.53	-0.485	0.628
Gravidity	2(1,3)	2(1,3)	-0.562	0.574
Parity	1(1,2)	1(0,2)	-1.937	0.053
Number of previous abortions	1(0,2)	1(0,1)	-3.588	<0.001
History of preterm birth	10(11.76)	22(5.30)	4.920	0.027
Gestational age at delivery	39.00(38.00, 40.00)	39.00(38.00, 40.00)	-0.765	0.444
Delivery type			2.665	0.264
Spontaneous vaginal delivery	43(50.59)	174(41.93)		
Assisted vaginal delivery	17(20.00)	83(20.00)		
Cesarean section	25(29.41)	158(38.07)		
Previous cesarean section	32(37.65)	83(20.00)	12.406	<0.001
Prior PPH	12(14.12)	18(4.34)	11.965	0.001
Anemia	29(34.12)	78(18.80)	9.847	0.002
Prolonged labor	18(21.18)	24(5.78)	21.726	<0.001
Placenta previa	11(12.94)	12(2.89)	16.236	<0.001
Macrosomia	12(14.12)	54(13.01)	0.075	0.784
Placental abruption	7(8.24)	7(1.69)	11.116	0.001
Placenta accreta	7(8.24)	6(1.45)	12.842	<0.001
Gestational hypertension	17(20.00)	62(14.94)	1.358	0.244
Gestational diabetes	19(22.35)	70(16.87)	1.451	0.228
Thyroid dysfunction	10(11.76)	45(10.84)	0.061	0.805
R (min)	8.48±1.23	8.45±1.59	−0.171	0.864
MA (mm)	50.15±4.16	49.98±4.17	-0.338	0.735
K (min)	1.85±0.36	1.82±0.38	-0.572	0.568
CI	2.00(1.40, 2.90	0.80(0.60, 1.10)	-10.919	<0.001
α-angle (°)	64.75±6.14	64.42±6.12	-0.458	0.647
TT (s)	18.24±1.86	18.16±1.92	-0.331	0.741
APTT (s)	29.14±3.52	29.08±3.41	-0.135	0.892
PT (s)	12.42±1.26	12.45±1.45	0.205	0.838
FIB (g/L)	1.90(1.60, 2.30)	3.14(2.47, 3.77)	-10.474	<0.001
D-D (μg/L)	0.50(0.40, 0.55)	0.37(0.29, 0.46)	-4.472	<0.001
AT-III (%)	78.48±15.73	91.45±20.38	6.559	<0.001

The occurrence of PPH among parturients was defined as the dependent variable (*Y*), and variables with statistically significant differences in Table-I were included as independent variables (*X*) for multivariate logistic regression analysis. Variable assignment was as follows: previous cesarean section(no = 0, yes = 1), history of preterm birth (no = 0, yes = 1), prior PPH (no = 0, yes = 1), anemia (no = 0, yes = 1), prolonged labor (no = 0, yes = 1), placenta previa (no = 0, yes = 1), placental abruption (no = 0, yes = 1), and placenta accreta (no = 0, yes = 1). The number of previous abortions, CI, FIB, D-D, and AT-III were continuous variables and were analyzed using their actual measurements. According to the results of logistic regression analysis (Table-II), previous cesarean section, prior PPH, anemia, CI, D-D and AT-III were independent risk factors for PPH (all *P <* 0.05), while FIB levels served as protective factors (all *P <* 0.05).

**Table-II:** Multivariate logistic regression analysis of risk factors associated with PPH in parturients.

Factors	β	SE	Wald χ²	OR	95% CI	P-value
Number of previous abortions	0.623	0.396	2.475	1.865	0.858~4.056	0.116
History of preterm birth	0.861	0.986	0.763	2.365	0.343~16.327	0.382
Previous cesarean section	1.133	0.51	4.947	3.106	1.144~8.431	0.026
Prior PPH	1.592	0.801	3.948	4.916	1.022~23.646	0.047
Anemia	1.069	0.52	4.226	2.911	1.051~8.065	0.04
Prolonged labor	1.271	0.699	3.306	3.565	0.906~14.034	0.069
Placenta previa	1.867	1.089	2.937	6.466	0.765~54.666	0.087
Placental abruption	1.568	1.023	2.35	4.799	0.646~35.647	0.125
Placenta accreta	0.346	1.812	0.036	1.414	0.041~49.293	0.848
CI	3.223	0.519	38.633	25.109	9.087~69.380	<0.001
FIB	-1.563	0.287	29.658	0.21	0.119~0.368	<0.001
D-D*10	4.073	1.667	5.97	1.503	1.084~2.083	0.015
AT-III	-0.038	0.012	9.769	0.963	0.940~0.986	0.002

**Fig.1:**
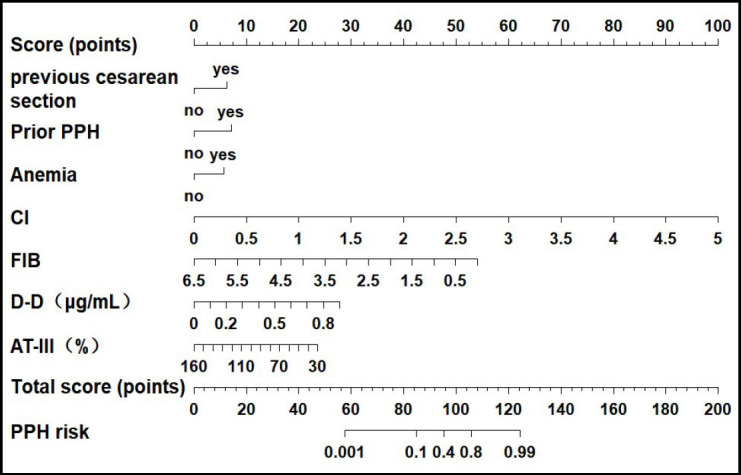
Nomogram model for predicting PPH risk in parturients.

This study continued to construct a risk prediction nomogram (Fig. 1) for PPH based on the six significant predictors(*i.e*., previous cesarean section, prior PPH, anemia, CI,D-D and AT-III). In clinical practice, corresponding values for each variable can be entered into the model to calculate an individualized risk score, with a higher total score corresponding to a higher predicted probability of PPH.

**Fig.2:**
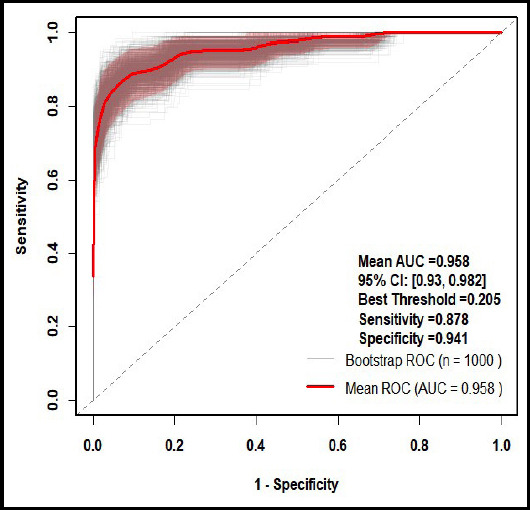
ROC curve.

**Fig.3:**
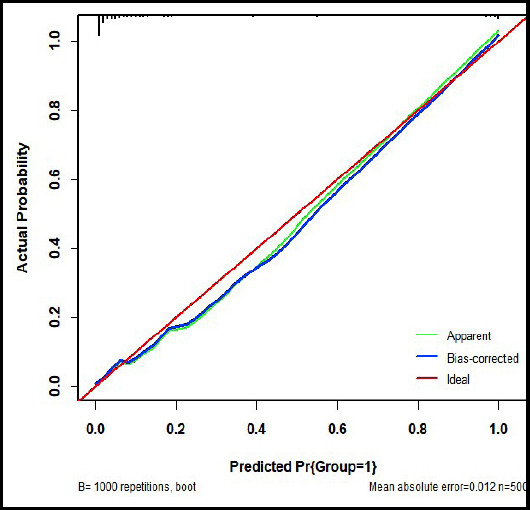
Calibration curve.

The predictive performance of the nomogram was internally validated using the Bootstrap resampling method (1,000 iterations),with an optimism-corrected bias of 0.007. The calibration curve slope was 0.978, and the Shrinkage coefficient was 0.978. The ROC curve (Fig.2) demonstrated that the nomogram achieved an AUC of 0.958 (95% CI: 0.93-0.982), with a sensitivity of 87.80% and a specificity of 91.40%, confirming that the model had excellent discriminatory power for predicting the occurrence of PPH. The calibration curve (Fig.3) showed that the predicted probabilities of PPH closely corresponded to the actual probabilities. The *Hosmer-Lemeshow* goodness-of-fit test yielded a χ² value of 11.417 (*P =* 0.1792).

## DISCUSSION

The present study retrospectively analyzed the clinical data from 500 parturients, and constructed a PPH risk prediction nomogram integrating TEG parameters and conventional coagulation parameters.^5^ Consequently, the constructed model offers a reliable, quantitative tool for the early identification of high-risk parturients as well as the formulation of individualized preventive and intervention strategies in clinical settings. Univariate analysis in this study revealed significantly statistical differences in the number of previous abortions, history of preterm birth, previous cesarean section, prior PPH, anemia, prolonged labor, placenta previa, placental abruption, placenta accreta, CI, FIB, D-D, and AT-III between the PPH and non-PPH groups, suggesting that these parameters may be potential influencing factors for the occurrence of PPH.^10^ Further multivariate logistic regression previous cesarean section, prior PPH, anemia, CI,D-D and AT-III were independent risk factors for PPH, while FIB served as protective factors. These findings are highly consistent with clinical observations and previous research reports.^11^ Previous cesarean section disrupts the continuity of uterine muscle fibers at the scar site, increasing the risk of uterine atony. Additionally, poor development of the decidua at the scar site may lead to placenta previa and placenta accreta.^12^

A history of PPH indicates that the parturient may have underlying coagulation abnormalities or uterine contractility dysfunction, which significantly increases the risk of recurrent PPH in subsequent deliveries.^13^ Anemia may reduce the blood’s oxygen-carrying capacity, impair uterine muscle contractility, and compromise hemostasis after delivery, thus predisposing to excessive bleeding.^14^

The CI serves as an integrated measure of overall coagulation status; a normal or slightly elevated CI may denote a balanced or hypercoagulable state, which may benefit the decrease of the risk of PPH.^15^ Similarly, Zou Y et al.^16^ elevated D-D level may be associated with enhanced fibrinolytic activity and disrupted coagulation-fibrinolysis balance, which predisposes to excessive bleeding. AT-III is the most important natural anticoagulant in the body. A decrease in its levels leads to insufficient anticoagulant capacity, disrupting the coagulation balance. The formation of microthrombi consumes large amounts of clotting factors and platelets, and secondary hyperfibrinolysis actually increases the risk of bleeding^17^. FIB, a key substrate in fibrin clot formation, is crucial for maintaining hemostatic integrity. Normal or elevated FIB levels can enhance fibrin network stability and improve clot firmness, thereby reducing bleeding risk. Conversely, decreased FIB concentrations may hinder fibrin formation and weaken hemostatic capacity, significantly increasing the likelihood of PPH.^18^ Altogether, TEG parameters and conventional coagulation parameters are mechanistically interconnected, and their combined assessment may provide a more comprehensive understanding of the complex relationship between coagulation and PPH risk.

According to the model validation results, the Bootstrap resampling method (1,000 iterations). The optimism-corrected bias was only 0.007, suggesting high internal reliability and minimal random error. The calibration slope of 0.978 and the Shrinkage coefficient of 0.978, together with the calibration curve showing close agreement between predicted and observed outcomes, demonstrated good model calibration. Furthermore, the *Hosmer-Lemeshow* goodness-of-fit test (χ² = 11.417, *P =* 0.1792) indicated that the predicted probabilities were highly consistent with actual PPH occurrences. ROC curve analysis further confirmed the robust predictive performance of this model, with an AUC of 0.958 (95% CI: 0.93-0.982), sensitivity of 87.80%, and specificity of 91.40%. In view of the above, the combined model achieved high accuracy in identifying women at risk for PPH. The high sensitivity ensures maximal detection of high-risk individuals, thereby reducing the likelihood of missed diagnoses, while the relatively high specificity minimizes false positives, avoiding unnecessary clinical interventions. Compared with the model based solely on conventional coagulation parameters, the integrated model combining TEG and conventional coagulation parameters exhibited distinct advantages. Conventional coagulation tests (*e.g*., PT, APTT, and FIB) reflect only isolated phases of the coagulation cascade or the activity of specific factors, providing a static and limited view of hemostatic function. In contrast, TEG offers a dynamic, whole-blood assessment, continuously monitoring multiple aspects of the coagulation process, including coagulation factor activation, platelet aggregation, fibrin polymerization, and fibrinolysis.^19^ Therefore, the integration of TEG and conventional coagulation parameters enables complementary strength. In other words, TEG captures the real-time dynamics of clot formation and dissolution, while conventional assays quantify coagulation factor levels. This comprehensive evaluation provides a more accurate and physiologically relevant representation of the maternal coagulation state, substantially enhancing the precision and reliability of PPH risk prediction.

### Limitations

First, this study was a single-center retrospective study with the recruitment of all participants from the same hospital, which may introduce selection bias and compromise the generalizability of the findings.

## CONCLUSIONS

In conclusion, this study developed a nomogram model integrating TEG parameters with conventional coagulation parameters to predict the risk of PPH in parturients. Previous abortions, history of preterm birth, previous cesarean section, prior PPH, anemia, prolonged labor, placenta previa, placental abruption, placenta accreta, coagulation index (CI), D-D, and AT-III are identified as independent risk factors; while FIB serve as protective factors. The combined model demonstrates excellent discrimination, calibration, and predictive performance.

### Recommendations:

Future multicenter, prospective studies with larger and more diverse populations are warranted to further validate and refine the applicability of this model. Second, this study performed only internal validation using bootstrap resampling, necessitating further verification to confirm the predictive accuracy of this model across different clinical settings and populations. Subsequent studies incorporating external validation are needed to confirm the robustness of the model and to enhance its clinical utility in guiding individualized PPH risk management.

### Authors’ Contributions:

**YZ:** Designed and did statistical analysis & editing of manuscript, is responsible for integrity of research.

**YZ** and **HW:** Conceived designed and helped in data collection.

**MW** and **YG:** Literature search, Did data collection and manuscript writing.

All authors have read and approved the final manuscript.
